# The Wheat GT Factor *TaGT2L1D* Negatively Regulates Drought Tolerance and Plant Development

**DOI:** 10.1038/srep27042

**Published:** 2016-06-01

**Authors:** Xin Zheng, Haipei Liu, Hongtao Ji, Youning Wang, Baodi Dong, Yunzhou Qiao, Mengyu Liu, Xia Li

**Affiliations:** 1 Center for Agricultural Resources Research, Institute of Genetics and Developmental Biology, Chinese Academy of Sciences, Shijiazhuang, Hebei 050021, P. R. China; 2 State Key Laboratory of Agricultural Microbiology, College of Plant Science and Technology, Huazhong Agricultural University, Wuhan, 430070, P. R. China; 3 University of Chinese Academy of Sciences, Beijing 100049, P. R. China; 4 School of Agriculture, Food and Wine, University of Adelaide, Waite Research Institute, Glen Osmond, SA 5064, Australia

## Abstract

GT factors are trihelix transcription factors that specifically regulate plant development and stress responses. Recently, several GT factors have been characterized in different plant species; however, little is known about the role of GT factors in wheat. Here, we show that *TaGT2L1A*, *TaGT2L1B*, and *TaGT2L1D* are highly homologous in hexaploid wheat, and are localized to wheat chromosomes 2A, 2B, and 2D, respectively. These *TaGT2L1* genes encode proteins containing two SANT domains and one central helix. All three homologs were ubiquitously expressed during wheat development and were responsive to osmotic stress. Functional analyses demonstrated that TaGT2L1D acts as a transcriptional repressor; it was able to suppress the expression of *AtSDD1* in *Arabidopsis* by binding directly to the GT3 box in its promoter that negatively regulates drought tolerance. *TaGT2L1D* overexpression markedly increased the number of stomata and reduced drought tolerance in *gtl1-3* plants. Notably, ectopic expression of *TaGT2L1D* also affected floral organ development and overall plant growth. These results demonstrate that TaGT2L1 is an ortholog of AtGTL1, and that it plays an evolutionarily conserved role in drought resistance by fine tuning stomatal density in wheat. Our data also highlight the role of TaGT2L1 in plant growth and development.

Drought is a major environmental factor limiting crop productivity and quality. The identification and application of genetic resources associated with dehydration responses and drought resistance are essential for screening and breeding new crops with increased quality and stress tolerance^1^. In recent decades, the signaling networks involved in abiotic stress responses and tolerance have been thoroughly defined and characterized. Transcription factor families, including WRKY, NAC, AP2/ERF, bHLH and MYB, play significant roles in regulating the expression of functional genes^2^,^3^,^4^,^5^,^6^,^7^. Thus, many studies have investigated the application of transcription factors to plant genetic engineering. In *Arabidopsis*, the NAC transcription factor ATAF1 influences abiotic and biotic stress responses, probably through a signaling pathway involving reactive oxygen species^8^; it also affects *DREB2A*-regulated water stress-responsive gene expression to modulate plant drought tolerance^9^. In crops, genetic engineering using various transcription factors has yielded plants with an increased ability to adapt to various stresses. For example, the overexpression of *TaPIMP1* (a MYB gene) significantly improved the drought tolerance capacity of transgenic wheat through the regulation of stress-responsive genes belonging to a hormone signaling pathway^10^. In rice, the drought-induced gene *OsWRKY47* has been shown to enhance drought tolerance, probably through the regulation of *CaBP* and *CRRSP*^11^.

Notably, GT factors, which belong to the trihelix transcription factor family, have also been shown to be crucial for plant abiotic stress responses^12^. GT factors contain a conserved trihelix DNA-binding domain, called a SANT (Swi 3 [switching-defective protein 3], Ada2 [adaptor 2], N-Cor [nuclear receptor co-repressor], TFIIB [transcription factor IIIB]) domain^13^. This domain binds specifically to the GT element located in a target gene’s promoter^14^,^15^,^16^. A genome-wide analysis revealed 319 putative trihelix genes in various plant species, including 30 members in *Arabidopsis*^12^,^17^, and functional analyses have revealed that GT factors are involved in multiple biological processes, including seed scattering, embryo development, floral organ morphogenesis, and most importantly, biotic and abiotic stress resistance in various plants^18^,^19^,^20^,^21^,^22^,^23^,^24^,^25^,^26^,^27^.

Stomata play a central role in plant drought responses by modulating transpiration and the uptake of carbon dioxide^28^. The plant hormone abscisic acid (ABA) initiates signaling pathways that control the drought response of guard cells^29^,^30^. Under drought conditions, the ABA content increases and triggers the activation of OPEN STOMATA 1 (SnRK2.6), a core regulatory component of the ABA signaling pathway; it phosphorylates and activates SLOW ANION CHANNEL ASSOCIATED 1, resulting in stomatal closure and plant tolerance to drought^31^,^32^. In addition to stomatal movement, stomatal size and density dramatically affect transpiration and carbon dioxide uptake in plants. It has been demonstrated that stomatal development is precisely controlled by a gene regulatory network^33^. In *Arabidopsis*, *SPEECHLESS* (*SPCH*), *MUTE*, and *FAMA* promote stomatal differentiation and development from meristemoid mother cells to guard cells, while the TOO MANY MOUTHS-ERECTA (TMM-ER) complex acts as a negative regulator^34^,^35^,^36^,^37^. The TMM-ER complex is activated by *STOMATAL DENSITY AND DISTRIBUTION1* (*SDD1*) encoding a subtilisin-like serine protease^38^,^39^, which causes the activation of a mitogen-activated protein kinase cascade and the phosphorylation of BREAKING OF ASYMMETRY IN THE STOMATAL LINEAGE and SPCH; this ultimately attenuates the basal signaling pathway underlying stomatal development^40^,^41^,^42^.

It is worth nothing that among the GT transcription factor family, GT-2 LIKE 1 (GTL1) has been identified as an important regulator of stomatal development in *Arabidopsis*^27^. Loss-of-function *gtl1* mutations result in a reduced number of stomata and lower stomatal density; as a result, *gtl1* mutant plants exhibit reduced stomatal conductance and leaf transpiration, leading to enhanced drought tolerance and water use efficiency^27^. Further analysis has shown that GTL1 binds directly to the GT3 box in the *SDD1* promoter, repressing its transcription; this in turn affects a key regulator of stomatal development, the TMM-ER complex, and, ultimately, modulates stomatal density^27^. Recently, it was demonstrated that the AtGTL1 ortholog in poplar, PtaGTL1, has the same biological functions as AtGTL1; *PtaGTL1* expression triggered by the *AtGTL1* promoter completely restored all of the defects observed in *gtl1* mutant (e.g., trichome size, stomatal development, water use efficiency, and drought tolerance). Importantly, PtaGTL1 binds not only to the *AtSDD1* promoter to regulate *AtSDD1* expression, it also binds to the promoter of the putative *PtaSDD1* gene, which encodes a protein with high identity to AtSDD1^43^. These results suggest that GTL1-mediated stomatal regulation and plant responses to water status represent a conserved mechanism in dicots.

Despite of our understanding of the role of GT factors in stomatal development in dicots, much less is known about the functions of GT factors in monocots. In rice, a total of 30 trihelix members have been identified^12^,^17^, and the downstream key regulators *OsSPCH2*, *OsFAMA*, and *OsMUTE* have been confirmed to have the same regulatory functions in stomatal development in monocots as they do in dicots^44^. These results suggest that GT factor-mediated mechanisms and downstream stomatal development might be conserved in monocots. This also led us to wonder whether GT factors function similarly to *AtGTL1* in regulating stomatal development and drought tolerance in bread wheat. In this report, we demonstrate that TaGT2L1 is an ortholog of AtGTL1. TaGT2L1 contains two SANT domains and is a member of the typical GT-2 superfamily. Functional characterization of TaGT2L1D revealed similar functions to AtGTL1 in stomatal density and drought tolerance. *TaGT2L1D* was found to be responsive to osmotic stress and acted as a transcriptional repressor of *AtSDD1* expression by binding to the GT3 box located in the *AtSDD1* promoter. Notably, *TaGT2L1* genes were ubiquitously expressed in multiple organs of wheat during plant development, with the highest level in floral organs during reproductive development; moreover, ectopic expression of *TaGT2L1D* resulted in abnormal plant growth and floral organ development. These results reveal the evolutionarily conserved role of *GT-2 LIKE* genes *in planta*, and they provide insight into the novel functions of trihelix transcription factors in bread wheat.

## Results

### TaGT2L1 proteins are GT-2 family members in bread wheat

**Figure 1:**
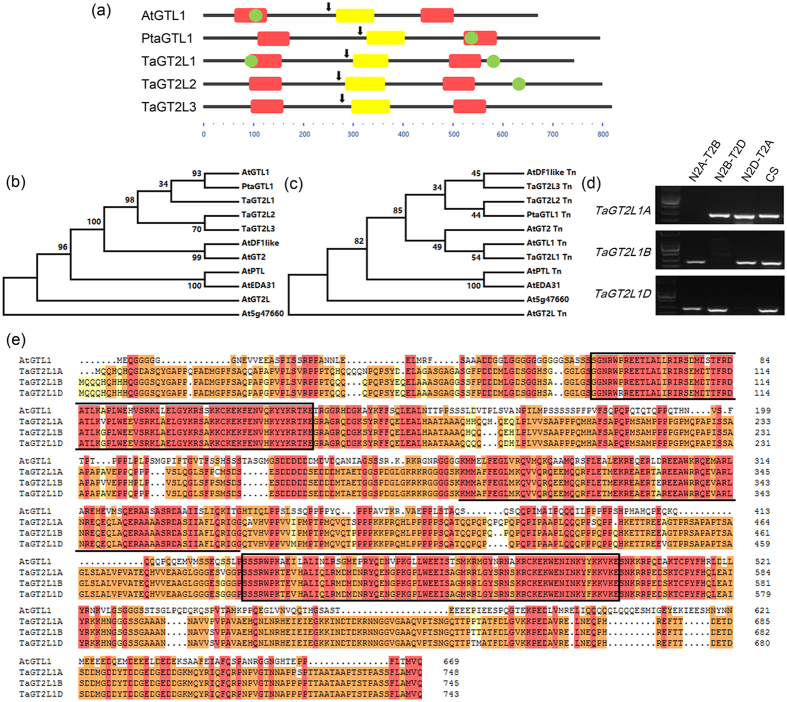
Identification of a bread wheat GT-2 family member, TaGT2L1. (**a**) Schematic diagrams of AtGTL1, PtaGTL1, TaGT2L1, TaGT2L2, and TaGT2L3. Red boxes highlight the predicted SANT domains; yellow boxes represent the predicted central helices; and green ovals indicate the positions of the predicted CaM-binding sites. Arrows indicate the positions of the predicted nuclear localization sequences. (**b**,**c**) Phylogenetic analysis of the *A. thaliana* GT-2 superfamily transcription factors, PtaGTL1, and TaGT2Ls. Tn indicates the N-terminal SANT domain. (**d**) PCR-basis analysis for chromosomal localization of the TaGT2L1s. N indicates nullisomic; T indicates tetrasomic. The letters A, B, and D in the line name indicate the three genomes of hexaploid wheat. CS indicates wild type Chinese Spring as a positive control. (**e**) Alignment of the TaGT2L1s with AtGTL1. The SANT domains are framed in black boxes; the conserved central helix is underlined.

To identify potential GT-2 LIKE genes, we performed a TBLASTN search of the *Triticum aestivum* transcription factor database and Ensembl Plants database using the sequences of AtGTL1 and PtaGTL1 as bait. Three predicted proteins containing two typical SANT domains were identified; they were named TaGT2L1, TaGT2L2, and TaGT2L3, respectively. To investigate whether these predicted proteins are encoded by functional genes in wheat, we isolated the full-length coding sequences of *TaGT2L1*, *TaGT2L2*, and *TaGT2L3* from hexaploid wheat and analysed their corresponding proteins. Similar to AtGTL1 and PtaGTL1, all three predicted proteins were found to contain an N-terminal SANT domain, a C-terminal SANT domain, a central helix, and a predicted nuclear localization signal; in addition, TaGT2L1 and TaGT2L2 were found to contain a predicted calmodulin (CaM)-binding site (Fig. 1a). It has been demonstrated that the SANT domain confers DNA-binding activity^15^, while the CaM-binding site enhances or reduces transcription factor activity^45^,^46^,^47^, and the nuclear localization signal directs proteins to the nucleus. Thus, it was deemed highly likely that TaGT2L1, TaGT2L2, and TaGT2L3 are nuclear proteins that function as transcription factors in wheat. A phylogenetic analysis of the full-length or N-terminus of the three homologs revealed that TaGT2L1 shares the highest identity with AtGTL1 (Fig. 1b,c). The results indicate that TaGT2L1 is a potential ortholog of AtGTL1. Thereafter, we focused our research on the biochemical and biological functions of TaGT2L1.

### The identification of three copies of wheat *TaGTL2L1*

Hexaploid wheat contains six sets of chromosomes, and each gene is usually present in three copies. To further characterize the functions of TaGT2L1, we first identified three homologous sequences from hexaploid wheat (cv. Shimai15). Based on the nucleotide sequence of *TaGT2L1*, we performed a BLAST search in the Ensembl Plants database. Three homologous sequences were located in wheat chromosomes 2A, 2B and 2D, respectively. Thus, the corresponding genes were named *TaGT2L1A*, *TaGT2L1B*, and *TaGT2L1D*. Specific primers based on the sequence information were designed and PCR cloning was performed to get the three homologous mixture. As these three homologous sequences are too similar in sequence to be separated using different primers, the PCR products were cloned into T-vector and sequenced to separate individual homolog (see Supplementary Fig. S1).

Next, we attempted to determine their locations on chromosomes A, B, and D using Chinese Spring nulli-tetrasomic (NT) lines. As shown in Fig. 1d, no PCR product was detected when PCR was performed using 2A nullisomic wheat genome DNA and *TaGT2L1A* specific primer as template and primer. However, *TaGT2L1B* and *TaGT2L1D* PCR products were detected using same template and corresponding primers. These results indicated that *TaGT2L1A* is located in the wheat chromosome 2A. Similar results also indicated that *TaGT2L1B* and *TaGT2L1D* is located in the wheat chromosome 2B and 2D, respectively. TaGT2L1A, TaGT2L1B, and TaGT2L1D were found to be highly conserved and to share a high level of identity, ranging from 93.89 to 97.08% (Fig. 1e). Notably, the N-terminal SANT domains of these TaGT2L1s were found to be highly identical to each other, and to be 85.51–86.96% identical to that of AtGTL1, suggesting that they exhibit similar DNA-binding activity and functionality.

### The *TaGT2L1s* were expressed in multiple organs and were responsive to osmotic stress

**Figure 2:**
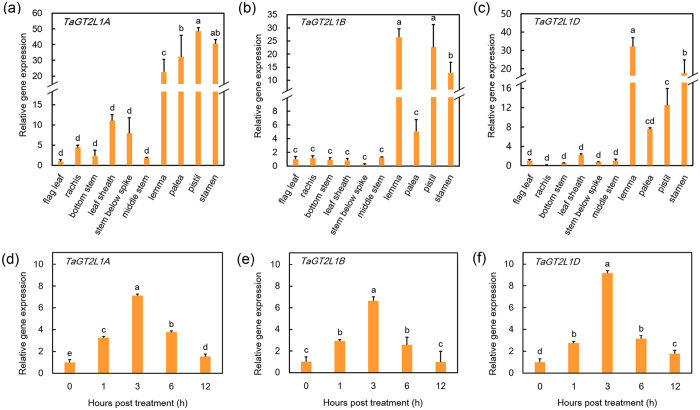
*TaGT2L1* expression patterns. Expression patterns of *TaGT2L1A* (**a**), *TaGT2L1B* (**b**), and *TaGT2L1D* (**c**) in different tissues were analysed at the anthesis stage. Changes in the expression patterns of *TaGT2L1A* (**d**), *TaGT2L1B* (**e**), and *TaGT2L1D* (**f**) in response to exposure to 20% (w/v) PEG6000 were analysed using one-week-old wheat seedling leaves. Total RNA was isolated and reverse-transcribed (n = 6). The qPCR was performed using *TaACTIN* gene as an internal reference. All values are the means ± standard deviations of three independent experiments. Different letters indicate significant differences (Student-Newman-Keuls test, P < 0.05).

To investigate whether *TaGT2L1A*, *TaGT2L1B*, and *TaGT2L1D* are involved in wheat development and are responsive to osmotic stress, we analysed their expression patterns using real-time quantitative PCR (qPCR). The *TaGT2L1s* showed universal expression in multiple organs at the anthesis stage in wheat (cv. Shimai15), indicating a universal function in wheat development (Fig. 2a–c). Interestingly, the expression levels of the *TaGT2L1s* in both pistil and stamen tissues were much higher than those in leaves and stems, although the exact expression levels of the three *TaGT2L1s* were different. These results suggest that *TaGT2L1s* play a major role in floral organ development in wheat plants.

Because AtGTL1 and PtaGTL1 mediate plant drought tolerance^27^,^43^, we examined whether *TaGT2L1A*, *TaGT2L1B*, and *TaGT2L1D* are responsive to osmotic stress. To this end, we performed qPCR and analysed the expression patterns of the *TaGT2L1s* in one-week-old wheat seedlings under osmotic stress. In the presence of 20% (w/v) PEG6000, all three homologs were sharply up-regulated and reached a peak within 3 h after treatment. Following the peak, all three homologs were down-regulated (Fig. 2d–f). The observation of similar expression patterns for *TaGT2L1A*, *TaGT2L1B*, and *TaGT2L1D* indicates that these homologs may play a similar role in the response of plants to osmotic stress.

It has been reported that *AtGTL1* was suppressed by drought stress^27^. The qPCR analysis showed that three weeks drought treatment obviously suppressed *TaGT2L1s* expression in wheat seedlings, while expression level of *TaGT2L1D* showed greatest reduction (see Supplementary Fig. S2), indicating its greater impact on regulating plant drought response. Thus, *TaGT2L1D* gene was selected for further functional analysis.

### TaGT2L1D is located in the nucleus

**Figure 3:**
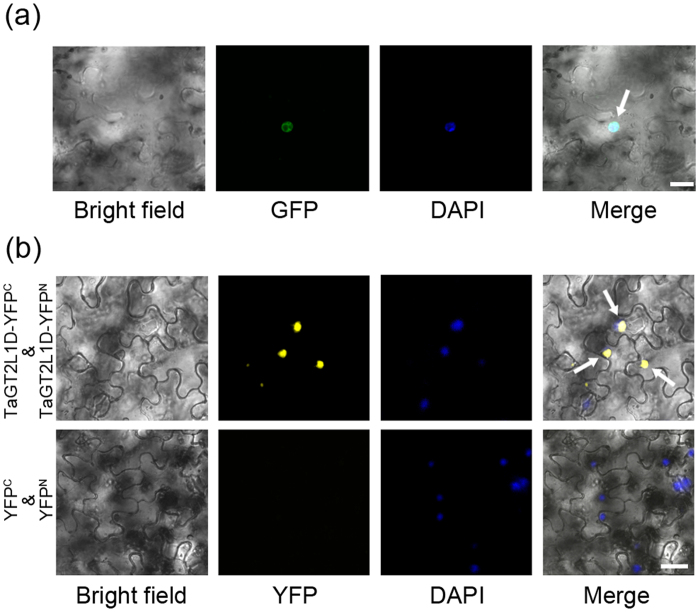
Subcellular localization of TaGT2L1D. (**a**) The construct harboring *GFP-TaGT2L1D* was transiently transformed to *N. benthamiana* leaves. (**b**) The construct harboring *TaGT2L1D-YFP*^*C*^ and *TaGT2L1D-YFP*^*N*^ were transiently co-transformed to *N. benthamiana* leaves. Constructs harboring *YFP*^*C*^ and *YFP*^*N*^ were used as negative control. All transformed leaves were stained with DAPI and then photographed by confocal microscopy. Bright field images show the complete epidermal cell profile in bright-field view, GFP/YFP or DAPI images show fluorescent signals in dark view, and Merge images show the overlay of three signals including bright field, GFP/YFP fluorescence and DAPI fluorescence. White arrows indicate the GFP or YFP fluorescence merged to the DAPI fluorescence. Scale bar = 50 μm.

Because TaGT2L1D has a putative nuclear localization signal and DNA-binding domains, we predicted that it should be localized to the nucleus. To confirm this prediction, we generated a construct harboring the full-length coding sequence of *TaGT2L1D* fused with the *green fluorescent protein* (*GFP*) gene under the control of the cauliflower mosaic virus (CaMV) 35S promoter and determined the subcellular localization of TaGT2L1D by transiently expressing the GFP-TaGT2L1D fusion protein in *Nicotiana benthamiana* leaf cells. As shown in Fig. 3a, strong GFP fluorescence was detected exclusively in the nucleus of *N. benthamiana* leaf cells; the nuclear localization of the fusion protein was confirmed by 4′,6-diamidino-2-phenylindole (DAPI) staining, which was used as a nuclear indicator.

It has been shown that the central helix facilitates the formation of a coiled coil^48^, which is associated with protein dimerization^12^. The structural similarities between TaGT2L1D, AtGTL1, and PtaGTL1 prompted us to test whether dimerization also occurs between TaGT2L1D proteins. To do so, we examined whether TaGT2L1D can interact with itself in plant cells via a bimolecular fluorescence complementation (BiFC) assay. Yellow fluorescent protein (YFP) signal should be detected in corresponding location when protein-protein interaction does exist. As expected, obvious YFP signal in the nucleus of *N. benthamiana* leaf cells was observed when TaGT2L1D-YFP^N^ with TaGT2L1D-YFP^C^ were co-expressed (Fig. 3b), which indicate that TaGT2L1D is located in the nucleus probably in a homodimer manner. These results demonstrate that TaGT2L1D may function as a homodimer in the nucleus.

### *TaGT2L1D* overexpression restores the trichome phenotype of *gtl1-3*

**Figure 4:**
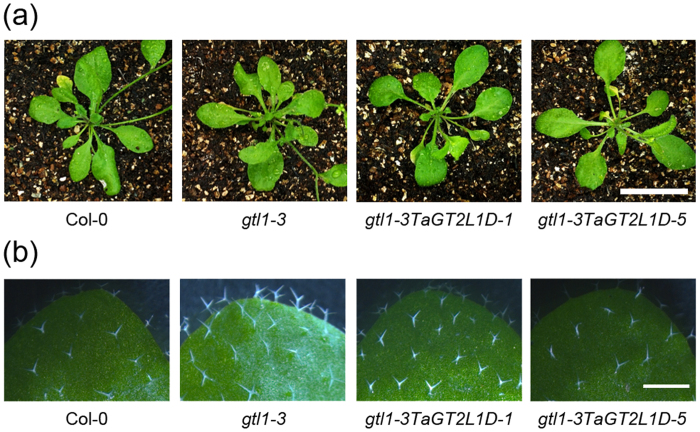
*TaGT2L1D* expression suppressed the leaf trichome phenotype of *gtl1-3*. (**a**) Photographs of rosette leaves in four-week-old Col-0, *gtl1-3*, *gtl1-3TaGT2L1D-1*, and *gtl1-3TaGT2L1D-5* plants were taken using a digital camera. Scale bar = 2 cm. (**b**) Trichomes on the adaxial surface of fully expanded rosette leaves from four-week-old plants. Photographs were taken using a dissecting microscope. Scale bar = 0.5 cm.

It has been shown that *AtGTL1* loss-of-function mutant *gtl1-3* reveals an increase in trichome size^19^. To analyse whether *TaGT2L1D* is a functional *AtGTL1* ortholog in wheat, we expressed a GFP-TaGT2L1D fusion protein under the control of the CaMV 35S promoter in *gtl1-3* mutant plants using the *Agrobacterium tumefaciens*-mediated floral-dip method. Five independent kanamycin-resistant T_1_ lines displayed a similar trichome size and whole plant size with wild-type Columbia-0 (Col-0); among the progeny of these plants, two T_3_ homozygous lines *gtl1-3TaGT2L1D-1* and *gtl1-3TaGT2L1D-5* were selected for further analysis. As shown in Fig. 4a, four-week-old *gtl1-3TaGT2L1D-1* and *gtl1-3TaGT2L1D-5* plants showed a similar phenotype in terms of rosette size to Col-0 and *gtl1-3* plants. The trichome size in *gtl1-3TaGT2L1D-1* and *gtl1-3TaGT2L1D-5* was completely restored to normal (Fig. 4b). RT-PCR analysis showed that *TaGT2L1D* transcripts were detected in the leaves of the transgenic lines, but not in Col-0 or *gtl1-3* plants (see Supplementary Fig. S3a).

Since *AtGTL1* can suppress the expression of *CELL CYCLE SWITCH 52 A1* (*CCS52A1*), leading to the termination of cell growth^49^, we questioned whether TaGT2L1D regulates trichome size in *Arabidopsis* through *CCS52A1*. To answer this question, we performed qPCR and examined the expression of *CCS52A1* in *gtl1-3TaGT2L1D-1* and *gtl1-3TaGT2L1D-5* lines. As expected, *CCS52A1* expression was significantly decreased in the transgenic lines compared with the high level of *CCS52A1* in the *gtl1-3* mutant (see Supplementary Fig. S3b), suggesting that *TaGT2L1D* functions similarly to *AtGTL1* in modulating trichome development.

### *TaGT2L1D* negatively regulates plant drought tolerance

**Figure 5:**
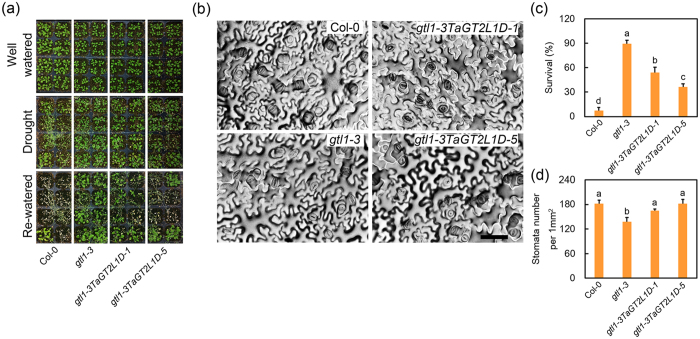
*TaGT2L1D* is involved in plant drought tolerance. (**a**) The plant water-deficit tolerance of two-week-old *gtl1-3TaGT2L1D-1*, and *gtl1-3TaGT2L1D-5* plants (16 h/8 h photoperiod) was evaluated. When the leaves of almost all of the plants were no longer turgid, the plants were re-watered. (**b**) An abaxial epidermal peel in the middle of the leaves from Col-0, *gtl1-3*, *gtl1-3TaGT2L1D-1*, and *gtl1-3TaGT2L1D-5* plants was printed and photographed to show the stomatal density. Scale bar = 50 μm. (**c**) The survival rate was measured after four days of re-watering. (**d**) The number of stomata per mm^2^ in the abaxial epidermis of fully expanded rosette leaves (four-week-old plants, 16 h/8 h photoperiod). All values are the means ± standard deviations of three independent experiments. Different letters indicate significant differences (Student-Newman-Keuls test, P < 0.05).

As mentioned, *AtGTL1* and *PtaGTL1* are involved in plant drought tolerance^27^,^43^. The fact that *TaGT2L1D* restored the trichome phenotype of *gtl1-3* suggested a similar role for *TaGT2L1D* in plant drought tolerance. To test this possibility, we performed a phenotypic analysis of *gtl1-3TaGT2L1D-1* and *gtl1-3TaGT2L1D-5* to determine whether *TaGT2L1D* expression could complement the drought-responsive phenotype of *gtl1-3*. The Col-0, *gtl1-3*, *gtl1-3TaGT2L1D-1*, and *gtl1-3TaGT2L1D-5* plants were watered thoroughly and uniformly for two weeks and then grown without watering for the next two weeks. When the leaves of almost all of the plants were no longer turgid, the plants were re-watered and the survival rate was measured after four days of re-watering (Fig. 5a). The survival rate of Col-0 was 12.5%, while that of *gtl1-3* was 90.6%, indicating that the loss of function of *GTL1* produced a better drought resistance phenotype in accordance with a previous report^27^. By contrast, the *gtl1-3TaGT2L1D-1* and *gtl1-3TaGT2L1D-5* lines exhibited substantially reduced survival rates (50.0 and 34.4%, respectively; Fig. 5c). *TaGT2L1D* expression partially restored the drought resistance of the *gtl1-3* mutant, suggesting a role for *TaGT2L1D* in plant drought responses.

Since *GTL1* regulates plant drought tolerance by modulating stomatal density^27^, the stomatal density in the rosette leaves of four-week-old Col-0, *gtl1-3*, *gtl1-3TaGT2L1D-1*, and *gtl1-3TaGT2L1D-5* plants were measured. The abaxial stomatal density in *gtl1-3* (138 ± 10 per mm^2^) was decreased by 24% compared with that in Col-0 (181 ± 9 per mm^2^), while the *gtl1-3TaGT2L1D-1* and *gtl1-3TaGT2L1D-5* lines showed significantly increased abaxial stomatal densities (165 ± 4 and 181 ± 10 per mm^2^, respectively) compared with *gtl1-3*; moreover, their stomatal densities were identical to that in wild-type (Fig. 5b,d).

It has been reported that GTL1 does not regulate stomatal aperture and ABA-induced stomatal closure^27^. Similarly, the stomatal aperture in the *gtl1-3TaGT2L1D-1* and *gtl1-3TaGT2L1D-5* lines under water-sufficient conditions was comparable to that in Col-0 and *gtl1-3* (see Supplementary Fig. S4a,b). Moreover, ABA treatment caused equivalent stomatal closure (see Supplementary Fig. S4a). These results indicate that *TaGT2L1D* is not involved in stomatal aperture or the response of plants to ABA, consistent with the role of *AtGTL1*^27^. Together, these data indicate that *TaGT2L1D* modulates plant drought tolerance by influencing stomatal density.

### TaGT2L1D *trans*-repressed *SDD1* expression in *Arabidopsis*

**Figure 6:**
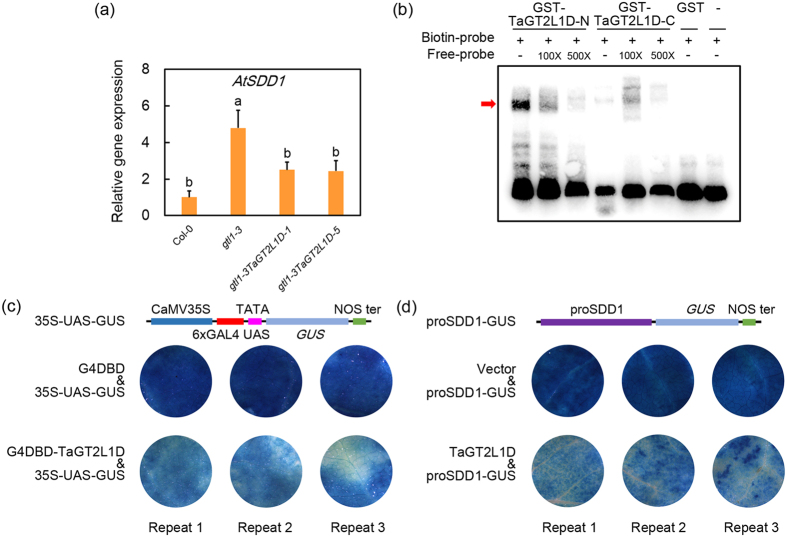
TaGT2L1D *trans*-represses *SDD1* expression in *Arabidopsis*. (**a**) *AtSDD1* expression in fully expanded rosette leaves from six-week-old Col-0, *gtl1-3*, *gtl1-3TaGT2L1D-1*, and *gtl1-3TaGT2L1D-5* plants (16 h/8 h photoperiod) was examined. Total RNA was isolated and reverse-transcribed (n = 6). The qPCR was performed using *AtACTIN* gene as an internal reference. All values are the means ± standard deviations of three independent experiments. Different letters indicate significant differences (Student-Newman-Keuls test, P < 0.05). (**b**) EMSAs were conducted using biotin-labeled DNA probes corresponding to a fragment of the *AtSDD1* promoter harboring the GT3 box (5′-GGTAAA-3′). The arrow indicates the interaction between the TaGT2L1D N-terminal fragment and the GT3 box probe. GST protein was used as a negative control. Three biological replications were performed. (**c**) The *trans*-repression activity of TaGT2L1D was tested in *N. benthamiana* leaves using a GAL4/UAS-based system. The G4DBD or G4DBD-TaGT2L1D was transiently co-expressed with the 35S-UAS-GUS reporter in *N. benthamiana* leaves, and the GUS staining was imaged. Three biological replications were performed. (**d**) TaGT2L1D represses the activity of the *SDD1* promoter in *N. benthamiana* leaves. The proSDD1-GUS reporter and GFP-TaGT2L1D were transiently co-expressed in *N. benthamiana* leaves and the GUS staining was imaged. GFP (Vector) was used as a negative control. Three biological replications were performed.

Since AtGTL1 and PtaGTL1 regulate stomatal development by *trans*-repressing the expression of *AtSDD1*^27^,^43^, we performed qPCR to analyse the effect of *TaGT2L1D* expression on *AtSDD1* expression in fully expanded leaves of *gtl1-3TaGT2L1D-1* and *gtl1-3TaGT2L1D-5* plants. Our results show that the up-regulation of *AtSDD1* in *gtl1-3* plants was markedly repressed by *TaGT2L1D* expression (Fig. 6a), indicating the negative regulatory effect of *TaGT2L1D* on *AtSDD1*. To verify that TaGT2L1D acts as a repressor, we performed a GAL4/UAS-based system assay for transcriptional activity^50^. TaGT2L1D fused with the GAL4 DNA-binding domain (G4DBD) was generated and co-transformed with a 35S-UAS-GUS reporter into *N. benthamiana* leaves. *GUS* (*β glucuronidase*) expression was repressed when TaGT2L1D was fused with the G4DBD (Fig. 6c), indicating that TaGT2L1D is a transcriptional repressor.

To determine whether TaGT2L1D *trans*-repressed *AtSDD1* expression directly, we generated a reporter construct harboring *proSDD1-GUS* and co-transformed it with *35S-GFP-TaGT2L1D* into *N. benthamiana* leaves. The *GUS* gene was well expressed when the *proSDD1-GUS* reporter was co-transformed with *35S-GFP*; by sharp contrast, *GUS* expression was repressed when *35S-GFP-TaGT2L1D* was co-transformed with *proSDD1-GUS* (Fig. 6d). AtGTL1 and PtaGTL1 repress *AtSDD1* expression by binding directly to the GT3 box (5′-GGTAAA-3′) located in the promoter of *AtSDD1*^27^,^43^. The fact that the N-terminal SANT domain of TaGT2L1D showed high identity with that of AtGTL1 suggested that TaGT2L1D regulates *AtSDD1* expression in the same way. To verify this, we purified the N and C-terminal SANT domain fragments of TaGT2L1D fused with a GST (glutathione S-transferase) tag (GST-TaGT2L1D-N and GST-TaGT2L1D-C, see Supplementary Fig. S5) to perform an electrophoretic mobility shift assay (EMSA) with a 28-nt *AtSDD1* promoter fragment that included the GT3 box. Mobility shift should be detected when protein-DNA interaction does exist. Our results show that TaGT2L1D-N, but not TaGT2L1D-C, caused a mobility shift in the biotin-labeled probe, and that unlabeled probe could eliminate the shift (Fig. 6b). These data indicates that TaGT2L1D modulates stomatal density and drought tolerance through the transcriptional repression of *AtSDD1* in *Arabidopsis*.

### *TaGT2L1D* is involved in plant development

**Figure 7:**
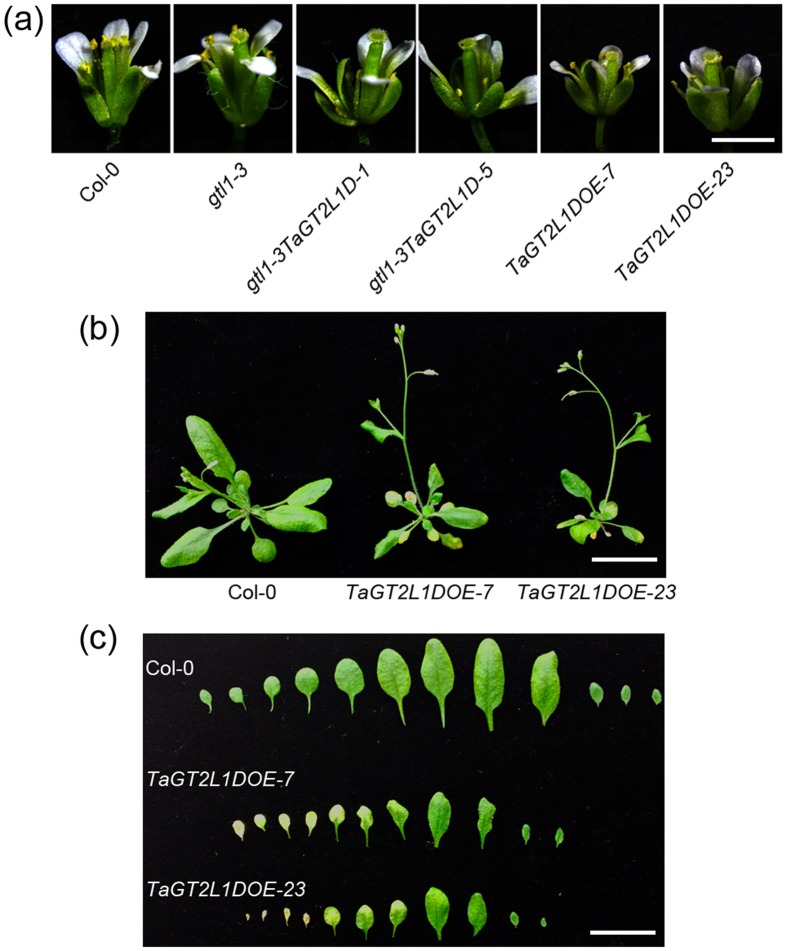
*TaGT2L1D* is involved in plant development. (**a**) Mature flowers from Col-0, *gtl1-3*, *gtl1-3TaGT2L1D-1*, *gtl1-3TaGT2L1D-5*, *TaGT2L1DOE-7*, *and TaGT2L1DOE-23* plants. Scale bar = 2 mm. (**b**) The size of rosette in four-week-old Col-0, *TaGT2L1DOE-7*, *and TaGT2L1DOE-23* plants (16 h/8 h photoperiod). Scale bar = 2 cm. (**c**) All rosette leaves were taken from four-week-old Col-0, *TaGT2L1DOE-7, and TaGT2L1DOE-23* plants (16 h/8 h photoperiod). Scale bar = 2 cm.

In addition to its conserved roles in trichome and stomatal development, we observed that ectopic *TaGT2L1D* expression in *gtl1-3* mutant plants caused developmental defects in the floral organs of the transgenic lines. The filaments of *gtl1-3TaGT2L1D-1* and *gtl1-3TaGT2L1D-5* lines were obviously shortened (Fig. 7a), leading to reduced fertility compared with Col-0 and *gtl1-3* plants. When *TaGT2L1D* was overexpressed in Col-0 under the control of the CaMV 35S promoter (see Supplementary Fig. S6), floral organ development in two overexpression lines, *TaGT2L1DOE-7* and *TaGT2L1DOE-23*, was visibly suppressed (Fig. 7a). This observation suggests that TaGT2L1D plays a role in floral organ development, which is favored by the high expression level of *TaGT2L1* in wheat floral organs.

Notably, lines *TaGT2L1DOE-7* and *TaGT2L1DOE-23* exhibited small rosettes compared with Col-0 (Fig. 7b) in accordance with the phenotype caused by the ectopic expression of *AtGTL1*^49^. Specifically, a significant decrease in rosette leaf size and slightly early senescence were observed (Fig. 7b,c). Moreover, lines *TaGT2L1DOE-7* and *TaGT2L1DOE-23* exhibited slightly early bolting (Fig. 7b). These results demonstrate that *TaGT2L1D* is involved in whole-plant development.

## Discussion

In recent years, the functions of GT factors in abiotic stress responses have been identified in various plant species. However, no functional analysis has been published regarding GT factors in bread wheat. Here, we characterized a drought-responsive gene, *TaGT2L1*, encoding a GT transcription factor in bread wheat. Our results demonstrate that *TaGT2L1* is an ortholog of AtGTL1 and that it plays an evolutionarily conserved roles in stomatal development and drought tolerance. Our data also indicate roles for *TaGT2L1* in plant growth and development, implying that this GT factor performs novel functions in bread wheat.

AtGTL1 is a transcription factor that belongs to the GT-2 superfamily of trihelix family proteins. It is a nuclear protein with DNA-binding activity that contains two SANT domains and one central helix^12^,^17^. AtGTL1 regulates stomatal development in *Arabidopsis* by repressing the expression of *AtSDD1* through binding to its promoter^27^. In this study, we identified orthologs of AtGTL1 (TaGT2L1s) in bread wheat; several pieces of evidence support this conclusion. First, the TaGT2L1s are structurally similar to AtGTL1, and they are evolutionarily close to AtGTL1. As a diploid plant, there is a single copy of the *GTL1* gene in *Arabidopsis*; in comparison, bread wheat is hexaploid and carries three *AtGTL1* homologs, *TaGT2L1A, TaGT2L1B*, and *TaGT2L1D* on chromosomes 2A, 2B, and 2D, respectively (Fig. 1d). Importantly, TaGT2L1 proteins contain two SANT domains and one central helix; further, the N-terminal SANT domain is highly conserved compared with that of AtGTL1 (Fig. 1e).

Second, we used TaGT2L1D to confirm that TaGT2L1 proteins are indeed localized to the nucleus (Fig. 3a). Although there is no classical nuclear localization signal in TaGT2L1D, its sequence does contain a predicted nuclear localization signal, ^284^KRKRGGGGSK^293^, which may mediate the nuclear localization of the protein. The predicted nuclear localization signal of TaGT2L1D is close to the central helix, which is believed to be associated with protein dimerization^12^. Notably, TaGT2L1D is able to interact with itself, which suggest a dimerization. It is likely that TaGT2L1D dimerization through the predicted nuclear localization signal in each monomer ensures the nuclear localization of the protein. Further analysis is needed to confirm the predicted dimerization of TaGT2L1s and to elucidate the role of this dimerization in the functions of these proteins.

Third, we found that TaGT2L1D has DNA binding activity and *trans*-repression activity, similar to AtGTL1 (Fig. 6b,c). Interestingly, a potential ethylene-responsive element binding factor-associated amphiphilic repression (EAR) motif, ^460^GLSLAL^465^, was identified in TaGT2L1D. It has been suggested that EAR motif-containing *trans*-repressors suppress the expression of their target genes through histone deacetylation at regulatory regions by physically interacting with co-repressors^51^,^52^,^53^. It is conceivable that TaGT2L1s repress the transcription of their target genes through a similar mechanism. The identification of these co-repressors and an analysis of chromatin modification (especially the histone acetylation status of candidate target genes) will reveal the molecular mechanism underlying the repression of gene expression by TaGT2L1s. Unexpectedly, there is no typical EAR motif in AtGTL1, indicating that AtGTL1 may utilize a different regulatory mechanism to repress the transcription of its target genes. To our knowledge, this is the third report to show that a GT factor can function as a *trans*-repressor (after GTL1 and ASR3). GTL1 is a *trans*-repressor that can suppress the expression of *SDD1* and *CCS52A1*^27^,^49^, while ASR3 is a *trans*-repressor that can suppress the gene expression via its ERF-associated amphiphilic repression binding activity^22^. The demonstrated functional similarity in transcriptional regulation supports the notion that TaGT2L1s are orthologs of AtGTL1.

The most important piece of evidence supporting the idea that TaGT2L1s are AtGTL1 orthologs is that TaGT2L1 proteins have similar biological functions to AtGTL1. The previous study has shown that *AtGTL1* was a drought-responsive gene and was suppressed by dehydration stress^27^. In this study, we found that *TaGT2L1s* were also down-regulated in leaves when wheat seedlings were exposed to drought stress (See Supplementary Fig. S2). The similar expression patterns of *TaGT2L1s* to *AtGTL1* in response to water stress indicate that these genes might play similar roles in drought tolerance of wheat. Further functional analysis results favor our hypothesis. Overexpression of *TaGT2L1D* complemented both the trichome size and stomatal density phenotypes of *gtl1-3* (Figs 4b and 5b). As a result, the stress tolerance of the *gtl1-3* mutant plants was largely restored by ectopic *TaGT2L1D* expression, confirming that *TaGT2L1D* modulates plant responses to water stress through the same regulatory mechanism as *AtGTL1*.

In addition, GTL1 binds to the GT3 box in the *SDD1* promoter and represses its expression, thereby inactivating the downstream TMM-ER complex and mitogen-activated protein kinase pathway to promote stomatal development^54^. In the present study, we found that TaGT2L1D was able to bind directly to the GT3 box in the *AtSDD1* promoter and suppress *AtSDD1* promoter activity (Fig. 6b,d). Like AtGTL1, TaGT2L1D appears to regulate plant drought resistance by modulating stomatal density. However, TaGT2L1D overexpression did not completely restore the drought tolerance of *gtl1-3* mutant plants (Fig. 5a). This may be explained by the functional discrepancy suggested by the structural differences between AtGTL1 and TaGT2L1D, including the EAR motif and amino acid sequences outside of the conserved SANT domains. These results imply that TaGT2L1 proteins have both a different regulatory mode, and novel biological roles in bread wheat.

Our results also confirm that *TaGT2L1* mediates plant growth and development in *Arabidopsis*. In addition to complementing of the trichome and stomata phenotypes of *gtl1-3*, *TaGT2L1D* overexpression suppressed filament elongation in the mutant (Fig. 7a), resulting in short siliques and reduced fertility in our transgenic lines. Taking into account the finding that the three *TaGT2L1* genes were expressed at high levels in wheat stamens during reproductive growth (Fig. 2a–c), it is possible that *TaGT2L1s* play crucial roles in filament elongation and reproduction in wheat. Identification of the targets of TaGT2L1s that modulate filament development will enhance our understanding of TaGT2L1s-mediated filament development and reproductive development in wheat. *AtGTL1* expression driven by the *ATML1* promoter was previously shown to cause a strong reduction in plant size^49^.It is supposed that GTL1 may control earlier progression of endocycles in non-trichome cells potentially by modulating the *CCS52A1* expression^49^. In this study, *TaGT2L1D* overexpression also suppressed the expression of *CCS52A1* and development of the whole plant in wild-type *Arabidopsis*, including floral organs (Fig. 7), indicating the function of *TaGT2L1D* in terminating cell growth. The universal expression of *TaGT2L1* in wheat supports its multiple biological functions in various developmental processes through *trans*-repression.

In summary, in this study we identified GT factors and orthologs of AtGTL1 in bread wheat. In addition to their roles in stomatal density regulation and plant drought tolerance, these TaGT2L1s modulate plant growth and development. Our findings provide novel insight into the functions of GT factors in drought tolerance and development in bread wheat. Further study of these *TaGT2L1s* and their underlying regulatory network will help us elucidate the genetic basis for development and drought tolerance in wheat, and it will promote the development of crop plants with increased stress tolerance.

## Materials and Methods

### Cloning and sequence analyses

Sequence information of *TaGT2L1s, TaGT2L2*, and *TaGT2L3* were obtained via a TBLASTN search of the plant transcription factors database (http://planttfdb.cbi.pku.edu.cn/) and Ensembl Plants database (http://plants.ensembl.org/index.html) using the sequences of AtGTL1 and PtaGTL1 as bait. Gene-specific primers were designed based on sequences from the plant transcription factors database and Ensembl Plants database and all primer sequences are listed in the Supplementary Table S1.

Total RNA was extracted from leaves of bread wheat (cv. Shimai15) using Trizol reagent (Invitrogen, Corp., Carlsbad, CA, USA) and treated with DNase I (Promega, Madison, WI, USA). A total of 5 μg total RNA was used for reverse transcription with a FastQuant RT Kit (Tiangen Biotech [Beijing] Co. Ltd., Beijing, China) according to the manufacturer’s protocol. The cDNA was used as template for cloning full-length coding sequences of *TaGT2L1s, TaGT2L2*, and *TaGT2L3*. PCR was performed using KOD FX DNA Polymerase (Toyobo, Tokyo, Japan) according to the manufacturer’s protocol. Positive PCR products were cloned into pMD19-T vector and independent clones were sequenced to identify individual sequence.

The amino acid sequences of TaGT2L1s, TaGT2L2, and TaGT2L3 were determined using the ExPASy Translate tool (http://web.expasy.org/translate/). The SANT domains were predicted through an NCBI conserved domain search (http://www.ncbi.nlm.nih.gov/Structure/cdd/wrpsb.cgi). The central helices were predicted using Jpred 4 (http://www.compbio.dundee.ac.uk/jpred4/index_up.html). The CaM-binding sites were predicted using a CaM-binding site search (http://calcium.uhnres.utoronto.ca/ctdb/ctdb/sequence.html). The position of the potential nuclear localization signal was predicted using NLStradamus (http://www.moseslab.csb.utoronto.ca/NLStradamus/). Sequence alignments were performed using Clustal Omega 1.2.1 (http://www.ebi.ac.uk/Tools/msa/clustalo/). Full-length amino acid sequences and N-terminal SANT domain sequences (according to Kaplan’s definition^12^, from four upstream of the first conserved tryptophan, to 12 downstream of the third conserved phenylalanine) were used to construct a phylogenetic tree by the neighbor-joining method with MEGA 6.05 and 1,000 bootstrap replicates.

### Chromosomal locations of the three *TaGT2L1* homologs

Three Chinese Spring NT lines, N2A-T2B (2A chromosome nullisomic line), N2B-T2D (2B chromosome nullisomic line), and N2D-T2A (2D chromosome nullisomic line) were used for the PCR-basis analysis of *TaGT2L1* chromosomal locations. Gene-specific primers for the three *TaGT2L1* homologs were designed based on the differences in their 3′-untranslated regions. PCR was performed to determine the chromosomal locations of the genes using wheat NT line genomic DNA as the template. The PCR conditions were as follows: 95 °C for 5 min; 35 cycles of 95 °C for 30 s, 61 °C for 30 s, and 72 °C for 30 s; and 72 °C for 10 min. The PCR products were separated by 2% agarose gel electrophoresis. All primer sequences are listed in the Supplementary Table S1.

### Gene expression analysis

Total RNA extraction and reverse transcription were performed as described previously. For tissue expression pattern analysis of *TaGT2L1s*, wheat plants (cv. Shimai15) were cultivated in the field under natural conditions with well irrigation. A variety of tissues, including leaf, stem, lemma, palea, pistil, stamen, and rachis, were sampled at the anthesis stage for the qPCR analysis. For examining *TaGT2L1s* expression pattern response to PEG treatment, wheat seedlings (cv. Shimai15) were grown in a controlled chamber with a 16 h/8 h photoperiod at 22 °C. One week after germination, the plants were exposed to 20% (w/v) PEG6000 solution for 1, 3, 6, and 12 h, after which their leaves were sampled for the qPCR analysis. For examining *TaGT2L1s* expression pattern in response to drought treatment, one-week-old wheat seedlings (cv. Shimai15) were grown in pots (10 cm in length, 10 cm in width, and 10 cm in height) in a controlled chamber with a 16 h/8 h photoperiod at 22 °C with or without watering for three weeks. Leaf samples were harvest at the same time for the qPCR analysis. For the gene expression analysis in *Arabidopsis*, plants were grown in pots (8 cm in length, 8 cm in width, and 8 cm in height) in a controlled chamber environment under a 16 h/8 h photoperiod at 22 °C and leaf samples were harvest for qPCR analysis.

The qPCR was performed in a Bio-Rad CFX Connect™ Real-Time PCR Detection System (Hercules, CA, USA) using a SYBR Green SuperReal PreMix Plus (Tiangen Biotech [Beijing] Co. Ltd.). The PCR parameters were: 95 °C for 15 min, followed by 40 cycles of 95 °C for 10 s and 61 °C for 30 s. Three biological replicates were used with three technical replicates for each biological replicate. Wheat *ACTIN* gene (GenBank accession number KC775780) and *Arabidopsis ACTIN* gene (GenBank accession number NM_179953) were used as internal references. Relative gene expression levels were detected using the 2^−ΔΔCT^ method^55^. All primer sequences are listed in the Supplementary Table S1.

### Subcellular localization and protein-protein interaction analysis

To verify the subcellular localization of TaGT2L1D, full-length coding sequence of *TaGT2L1D* were amplified by PCR and then cloned into pTF101-GFP vector with *Xma*I to create a *35S-GFP*-*TaGT2L1D* fusion. The plasmid was introduced into *A. tumefaciens* (strain EHA105). The infiltration of *N. benthamiana* was performed as described previously^56^. Briefly, transformed *A. tumefaciens* cells were grown at 28 °C in Luria-Bertani liquid medium and then incubated at room temperature for 3 h, followed by the infiltration into the abaxial air spaces of *N. benthamiana* leaves. Epidermal cell layers were stained with DAPI and assayed for fluorescence 3 d after infiltration. GFP and DAPI fluorescence were observed with a confocal microscope (Leica TCS-SP8; Leica Microsystems, Wetzlar, Germany).

To verify the potential interaction between TaGT2L1D itself in plant cells, a widely used BiFC assay was performed as described previously^57^. The full-length coding sequence of *TaGT2L1D* was cloned into pDONR207 using BP Clonase Enzyme (Invitrogen Corp.) and then recombined in the Gateway-compatible destination vectors pEarleyGate201-YN (N-terminal YFP) and pEarleyGate202-YC (C-terminal YFP) using LR Clonase Enzyme (Invitrogen Corp.) according to the manufacturer’s protocol. The resulting plasmids were transiently co-transformed into *N. benthamiana* leaves using *A. tumefaciens* (strain EHA105) as described previously. The reconstituted YFP fluorescence was observed with a confocal microscope (Leica TCS-SP8; Leica Microsystems). Empty vectors were used as a negative control.

These experiments were performed with three biological replications. All primer sequences are listed in the Supplementary Table S1.

### Generation of *TaGT2L1D* transgenic *Arabidopsis* plants

*Arabidopsis thaliana* ecotype Col-0 and *GTL1* loss-of-function mutant *gtl1-3* (SALK_005966) plants were used to generate *TaGT2L1D* transgenic plants. The plants were germinated on Murashige and Skoog (MS; pH 5.7) medium containing 1% (w/v) sucrose. One-week-old seedlings were transferred to pots and grown in a controlled chamber environment under a 16 h/8 h photoperiod at 22 °C. The full-length coding sequence of *TaGT2L1D* was amplified by PCR and cloned into the *Eco*RI*/Xba*I sites in pEZR(K)-LC vector to put gene under the control of the CaMV 35S promoter. The resulting plasmid was introduced into *A. tumefaciens* (strain EHA105), and transgenic *Arabidopsis* plants were generated using the *A. tumefaciens*-mediated floral dip method. Homozygous T_3_ plants were screened on plates containing MS medium with 75 mg/L of kanamycin. *TaGT2L1D* expression in transgenic *Arabidopsis* plants was examined by Semi-quantitative RT-PCR, which was conducted as follows: 95 °C for 5 min; 30 cycles of 95 °C for 30 s, 57 °C for 30 s, and 72 °C for 30 s; and 72 °C for 10 min. The sequences of the primers used are listed in the Supplementary Table S1.

### Drought treatment, stomatal density and stomatal aperture measurement

Col-0, *gtl1-3*, *gtl1-3TaGT2L1D-1* and *gtl1-3TaGT2L1D-5* plants were cultivated in pots (50 cm in length, 25 cm in width, and 5 cm in height) under controlled chamber environment with a 16 h/8 h photoperiod at 22 °C. For drought stress treatment, plants were grown with sufficient watering for two weeks after germination. Water was withheld for the next two weeks. When the leaves of almost all of the plants were no longer turgid, the plants were re-watered and the survival rate was measured after four days of re-watering.

For stomatal density measurement, fully expanded four-week-old leaves from Col-0, *gtl1-3*, *gtl1-3TaGT2L1D-1* and *gtl1-3TaGT2L1D-5* lines were used. The number of stomata was determined in a 0.144 mm^2^ area using an abaxial epidermal peel taken from the middle of each leaf. Four areas per leaf were measured, and six leaves from each different individuals were analysed. The epidermis was printed using Super Glue (Ontario, CA) and the imprint on the glass slide was observed with a confocal microscope (Leica TCS-SP8; Leica Microsystems).

The stomatal aperture was analysed using abaxial epidermal peels of fully expanded leaves according to a previous report^58^. For ABA treatment, fully expanded four-week-old leaves from Col-0, *gtl1-3*, *gtl1-3TaGT2L1D-1* and *gtl1-3TaGT2L1D-5* plants were incubated in a buffer solution (20 mM KCl, 5 mM MES-KOH, and 1 mM CaCl_2_, pH 6.15) for 2 h to open stomata, and then incubated with 20 μM ABA for 1 h. The aperture width of each stomatal pore was determined.

### Transcriptional activity analysis of TaGT2L1D

An effective GAL4/UAS-based system was performed as described previously^59^ to assess the transcriptional activity of TaGT2L1D in plant. The coding sequence of *TaGT2L1D* was cloned into *EcoR*I/*Sal*I of pYF503 vector to produce a *G4DBD-TaGT2L1D* fusion. The *G4DBD* and *G4DBD-TaGT2L1D* fragments were cloned into pDONR207 and then recombined in the Gateway-compatible destination vector pGWB2 to generate the effector constructs. The *35S-UAS-GUS* reporter plasmid and effector constructs were then transformed into *A. tumefaciens* (strain EHA105). The two effectors were then transiently co-transformed with the reporter into *N. benthamiana* leaves. After 24 h of incubation in the dark and 72 h of incubation in the light, the leaves were subjected to histochemical GUS staining as described previously^60^. Leaf samples were incubated in freshly prepared buffer containing 5-bromo-4-chloro-3-indolyl-β-D-glucuronic acid (X-gluc) overnight, followed by the cleanup with 75% ethanol.

For the repression of *AtSDD1* promoter activity by TaGT2L1D, The promoter of *AtSDD1* (1987-bp upstream of translation initiation site) was cloned into *Pst*I/*BamH*I of pCambia3301 vector to generate a *proSDD1-GUS* reporter construct. pTF101-GFP-TaGT2L1D was used as a positive effector, while pTF101-GFP was used as a negative control effector. A transient co-expression assay was performed in *N. benthamiana* leaves, followed by histochemical GUS staining as described previously.

These experiments were performed with three biological replications. All primer sequences are listed in the Supplementary Table S1.

### EMSAs

To determine DNA binding activity and the specificity of TaGT2L1D *in vitro*, EMSAs were performed using a LightShift Chemiluminescent EMSA Kit (Thermo Fisher Scientific, Waltham, MA, USA) according to the manufacturer’s protocol. Protein samples were designed to be fused with GST tag for purification process. For the GST-TaGT2L1D-N and GST-TaGT2L1D-C fusion proteins, the 5′ end 513-bp fragment and 3′ end 831-bp fragment were amplified and cloned using *Eco*RI and *Sal*I into pGEX-4T-1, respectively. The recombinant proteins were expressed in *Escherichia coli* and purified using amylose resin (New England Biolabs, Ipswich, MA, USA) according to the manufacturer’s protocol. For the nucleotide probes, an oligonucleotide containing the GT3 box (5′-GGTAAA-3′) from the *AtSDD1* promoter was synthesized and labeled with biotin at its 5′ end (Invitrogen Corp.). After 30 min of incubation at room temperature, the protein-probe mixture was separated on a 6% polyacrylamide gel and transplanted to a Biodyne B nylon membrane (Pall Corp., Port Washington, NY, USA). Migration of the biotin-labeled probes was detected by chemiluminescence according to the manufacturer’s protocol. This experiment was performed three times. All primer and probe sequences are listed in the Supplementary Table S1.

### Statistics

Statistical analysis was performed using the SPSS 21.0 software. Unpaired two-tailed t test was used for comparison between drought treatment group and control treatment group in the *TaGT2L1s* expression analysis. One-way analysis of variance (one-way ANOVA) followed by Student-Newman-Keuls test (as a post hoc test) was used for the rest of the statistical comparisons. All data are presented as mean ± standard deviations. P < 0.05 was considered as significant in the statistical analysis.

## Additional Information

**Accession codes**: Sequences of the genes in this paper may be found in the GenBank database library under the following accession numbers: KU559336 (*TaGT2L1*), AK336250 (*TaGT2L2*), AK333756 (*TaGT2L3*), KC775780 (*TaACTIN*), NM_179953 (*AtACTIN*), NM_100292 (*SDD1*), AY099585 (*CCS52A1*).

**How to cite this article**: Zheng, X. *et al*. The Wheat GT Factor *TaGT2L1D* Negatively Regulates Drought Tolerance and Plant Development. *Sci. Rep*. **6**, 27042; doi: 10.1038/srep27042 (2016).

## Supplementary Material

Supplementary Information
