# Supplementary material for: The Wheat GT Factor TaGT2L1D Negatively Regulates Drought Tolerance and Plant Development
Source: Sci Rep. 2016 Jun 1;6:27042. doi: 10.1038/srep27042 (PMC4887992; doi:10.1038/srep27042)
Supplement: Supplementary Information [file srep27042-s1.pdf]

Title

The Wheat GT factor *TaGT2L1D* Negatively Regulates Drought Tolerance and Plant Development

Authors and addresses

Xin Zheng[1, 2], Haipei Liu[3], Hongtao Ji[4], Youning Wang[4], Baodi Dong[1], Yunzhou Qiao[1], Mengyu Liu[1*], Xia Li[4*]

[1]Center for Agricultural Resources Research, Institute of Genetics and Developmental Biology, Chinese Academy of Sciences, Shijiazhuang, Hebei 050021, P.R. China

[2]University of Chinese Academy of Sciences, Beijing 100049, P.R. China

[3]School of Agriculture, Food and Wine, University of Adelaide, Waite Research Institute, Glen Osmond, SA 5064, Australia

[4]State Key Laboratory of Agricultural Microbiology, College of Plant Science and Technology, Huazhong Agricultural University, Wuhan, 430070, P.R. China

[*]Corresponding author

Xia Li, Ph.D., Professor

State Key Laboratory of Agricultural Microbiology, College of Plant Science and Technology, Huazhong Agricultural University, Wuhan 430070, P.R. China

E-mail: xli@mail.hzau.edu.cn

Telephone number: 86-311-85871744

[*]Co-corresponding author:

Mengyu Liu, Ph.D., Professor

Center for Agricultural Resources Research, Institute of Genetics and Developmental Biology, Chinese Academy of Sciences, Shijiazhuang, Hebei 050021, P.R. China

E-mail: mengyuliu@sjziam.ac.cn

Telephone number: 86-311-85871562

E-mail address of each author

Xin Zheng: claudezx@gmail.com

Haipei Liu: haipei.liu@adelaide.edu.au

Hongtao Ji: htji@mail.hzau.edu.cn

Youning Wang: youningwang@mail.hzau.edu.cn

Baodi Dong: dongbaodi@126.com

Yunzhou Qiao: yunzhouqiao@hotmail.com

Mengyu Liu: mengyuliu@sjziam.ac.cn

Xia Li: xli@mail.hzau.edu.cn

Supplementary Information

Supplementary Table S1 The sequences of the primers and probes used in this study

Supplementary Fig. S1 The nucleotide sequences of *TaGT2L1A*, *TaGT2L1B*, and *TaGT2L1D*

Supplementary Fig. S2 Drought treatment suppressed the expression of *TaGT2L1A*, *TaGT2L1B*, and *TaGT2L1D*

Supplementary Fig. S3 Expression levels of *TaGT2L1D* and *AtCCS52A1* in Col-0, *gtl1-3*, and *gtl1-3TaGT2L1D* lines

Supplementary Fig. S4 Stomatal aperture in wild Col-0, *gtl1-3*, and *gtl1-3TaGT2L1D* lines

Supplementary Fig. S5 Expression and purification of GST-TaGT2L1D-N and GST-TaGT2L1D-C proteins

Supplementary Fig. S6 Expression level of *TaGT2L1D* in the *TaGT2L1DOE* lines

Supplementary Table S1 The sequences of the primers and probes used in this study.

**Application**	**Name**	**Sequence (5′-3′)**
Full-length CDS	TaGT2L1-FL-F	CCAACCACCTGTTGCACATACAGA
	TaGT2L1-FL-R	GCATGGAACGGTGACTCGGTG
	TaGT2L2-FL-F	GCGACTGAGGAGGTGAGGG
	TaGT2L2-FL-R	CGACGATTTGGGCTTTCTT
	TaGT2L3-FL-F	GTGCACCCAGTGTGTACTA
	TaGT2L3-FL-R	GGTGGATCTACTGGACCAT
Chromosomal locations	TaGT2L1-chrA-F	GCCATCACATCACACCATCGAT
	TaGT2L1-chrA-R	AATTCTTGGCCTTAACACGAGG
	TaGT2L1-chrB-F	TCCAGCGCATCGGTGGTC
	TaGT2L1-chrB-R	GCAGCTGGGATGGGTTGC
	TaGT2L1-chrD-F	AGTTCCAGAGGCCAAACCCG
	TaGT2L1-chrD-R	GGTGACTCGGTGATGGTGGAATGA
qPCR	TaGT2L1A-q-F	GCCATCACATCACACCATCGAT
	TaGT2L1A-q-R	AATTCTTGGCCTTAACACGAGG
	TaGT2L1B-q-F	TCCAGCGCATCGGTGGTC
	TaGT2L1B-q-R	GCAGCTGGGATGGGTTGC
	TaGT2L1D-q-F	AGTTCCAGAGGCCAAACCCG
	TaGT2L1D-q-R	GGTGACTCGGTGATGGTGGAATGA
	TaACTIN-q-F	CCTCTCTGCGCCAATCGT
	TaACTIN-q-R	TCAGCCGAGCGGGAAATTGT
	AtSDD1-q-F	GCCATTGGAACATTCCGAGC
	AtSDD1-q-R	CTGACCACAGCGGGAAATCT
	AtCCS52A1-q-F	CACGCTGCAAGAGAACAAGA
	AtCCS52A1-q-R	ACCACTTGAGTCCGCATACC
	AtACTIN-q-F	GTCTGGATTGGAGGGTC
	AtACTIN-q-R	TGAGAAATGGTCGGAAA
RT-PCR	AtACTIN-RT-F	GTCTGGATTGGAGGGTC
	AtACTIN-RT-R	TGAGAAATGGTCGGAAA
	TaGT2L1D-RT-F	AAGCAACGGGCAGACAAC
	TaGT2L1D-RT-R	CGTCAGTGTAGTCATCGCC
Subcellular location	TaGT2L1D-Xma1-F	TCCCCCCGGGATGCAGCAGCAGCATCAGCAC
	TaGT2L1D-Xma1-R	TCCCCCCGGGCTACTGAACCATGGCGAGGAAG G
BiFC assay	TaGT2L1D-gateway-F	GGGGACAAGTTTGTACAAAAAAGCAGGCTTCA TGCAGCAGCAGCATCAGC
	TaGT2L1D-gateway-R	GGGGACCACTTTGTACAAGAAAGCTGGGTCCT GAACCATGGCGAGGAAG
Transgenic *Arabidopsis*	TaGT2L1D-EcoR1-F	CCGGAATTCATGCAGCAGCAGCATCAGCAC
	TaGT2L1D-Xba1-R	CTAGTCTAGACTACTGAACCATGGCGAGGAAG G

EMSAs	TaGT2L1D-N-EcoR1-F	CCGGAATTCATGCAGCAGCAGCATCAGCAC
	TaGT2L1D-N-Sal1-R	ACGCGTCGACCTAGAAGAAGCGGTAGCTCTTGCC
	TaGT2L1D-C-EcoR1-F	CCGGAATTCGTCGCCACGGAGCAGCACG
	TaGT2L1D-C-Sal1-R	ACGCGTCGACCTACTGAACCATGGCGAGGAAGG
	SDD1-GT3box-F-probe	TTCTTTGGCTTGGTAAAACTTCAATGGA
	SDD1-GT3box-R-probe	TCCATTGAAGTTTTACCAAGCCAAAGAA
Trans-repression assay	TaGT2L1D-pYF503-EcoR1-F	CCGGAATTCATGCAGCAGCAGCATCAGC
	TaGT2L1D-pYF503-Sal1-R	ACGCGTCGACCTACTGAACCATGGCGAGGA
	GAL4-TaGT2L1D-F	GGGGACAAGTTTGTACAAAAAAGCAGGCTATGAAGCTACTGTCTTCTATCG
	GAL4-TaGT2L1D-R	GGGGACCACTTTGTACAAGAAAGCTGGGTCTACTGAACCATGGCGAGGA
	proSDD1-Pst1-F	AAAACTGCAGCGAACGGGAAGCGGAAAG
	proSDD1-BamH1-R	CGCGGATCCTGGAGAGAGTTAAAAAAAGGAGTTG

```
TaGT2L1A        ---------ATGCAGCAGCACCAGCACCAGGGTGACGCGTCTCAGTACGGGGCGCCGCCG
TaGT2L1B        ATGCAGCAGCAGCATCAGCACCACCACCAGGGTGGCGGGTCTCAGTACGGGGC---GCCG
TaGT2L1D        ATGCAGCAGCAGCATCAGCACCACCACCAGGGTGGCGGGTCTCAGTACGGGGC---GCCG
                           *** ******** ********** ** ***************   ****

TaGT2L1A        CAGCCGGCGGACATGGGGCCGTTCTCCGCGCAGCAGGCTCCGGCCCCTGTGCCGCTGAGC
TaGT2L1B        CCGCCGGCGGACATGGGGCCGTTCTCCGCGCAGCCGGCTCCGGGCCCGGTGCCGCTGAGC
TaGT2L1D        CCGCCGGCGGACATGGGGCCGTTCTCCGCGCAGCCGGCTCCGGGCCCGGTGCCGCTGAGC
                * ******************************** ******** *** ************

TaGT2L1A        GTCCGGCCACCGCCGACCCAGCACCAGCAGCAGCAGAACCCGCAGCCGAGCTACGACGAA
TaGT2L1B        GTCCGGCCACCGCCGACGCAGCAGCAGCAGCCA------CAGCCGAGCTACGAACAGGAA
TaGT2L1D        GTCCGGCCACCGCCGACGCAGCAGCAGCAGCCA------CAGCCGAGCTACGAACAGGAA
                ***************** ***** *******        * ** *     * *  * ***

TaGT2L1A        TTGGCCGGCGCGTCGGGCGCCGGCGCCAGCGGCTTCCCCGACGACGACATGCTGGGCGAC
TaGT2L1B        TTGGCCGCCGCGTCGGGCGCCGGCGGAAGCAGCTTCCCCGACGACGACATGCTGGGCGAC
TaGT2L1D        TTGGCCGCCGCGTCGGGCGCCGGCGGAAGCAGCTTCCCCGACGACGACATGCTGGGCGAC
                ******* *****************  *** *****************************

TaGT2L1A        TCCGGCGGGCACAGCGGCGGCGGGCTGGGGTCGGGCGGCAACCGGTGGCCGCGGGAGGAG
TaGT2L1B        TCCGGCGGCCACAGCGCCGGCGGGCTGGGGTCGGGCGGCAACCGGTGGCCGCGGGAGGAG
TaGT2L1D        TCCGGCGGCCACAGCGCCGGCGGGCTGGGGTCGGGCGGCAACCGGTGGCGGCGGGAGGAG
                ******** ******* ******************************** **********

TaGT2L1A        ACGCTGGCCCTCATCAGGATCCGGTCGGAGATGGACACCACCTTCCGCGACGCCACCCTC
TaGT2L1B        ACGCTGGCCCTCATCAGGATCCGGTCGGAGATGGACACCACCTTCCGCGACGCCACCCTC
TaGT2L1D        ACGCTGGCCCTCATCAGGATCCGGTCGGAGATGGACACCACCTTCCGCGACGCCACCCTC
                ************************************************************

TaGT2L1A        AAGGTCCCCCTCTGGGAGGAGGTCTCCAGGAAGCTTGCGGAGCTGGGCTACAAGAGGAGC
TaGT2L1B        AAGGGCCCCCTCTGGGAGGAGGTCTCCAGGAAGCTTGCGGAGCTGGGCTACAAGAGGAGC
TaGT2L1D        AAGGGCCCCCTCTGGGAGGAGGTCTCCAGGAAGCTTGCGGAGCTGGGCTACAAGAGGAGC
                **** *******************************************************

TaGT2L1A        GCCAAGAAGTGCAAGGAGAAGTTCGAGAACGTGCACAAGTACTACAAGCGCACCAAGGAG
TaGT2L1B        GCCAAGAAGTGCAAGGAGAAGTTCGAGAACGTGCACAAGTACTACAAGCGCACCAAGGAG
TaGT2L1D        GCCAAGAAGTGCAAGGAGAAGTTCGAGAACGTGCACAAGTACTACAAGCGCACCAAGGAG
                ************************************************************

TaGT2L1A        GGCCGCGCCGGCCGGCAGGACGGCAAGAGCTACCGCTTCTTCCAGGAGCTCGAGGCACTG
TaGT2L1B        GGCCGGGCCGGCCGGCAGGACGGCAAGAGCTACCGCTTCTTCCAGGAGCTCGAGGCGCTG
TaGT2L1D        GGCCGGGCCGGCAGGCAGGACGGCAAGAGCTACCGCTTCTTCCAGGAACTCGAGGCGCTG
```

```
                 ***** ****** ********************************** ******** ***

TaGT2L1A         CACGCCGCCACCGCCGCCGCGCAGCACCACCAGCAGCACCAGGAGCAGTTGCCTCTCGTC
TaGT2L1B         CACGCCGCCACCGCAGCCGCGCAGCACCAG------CAGCAGGAGCACTTGCCGCTGGTC
TaGT2L1D         CACGCCGCCACCGCCGCCGCGCAGCAGCAG------CAGCAGGACCACTTGCCGCTGGTC
                 ************** *********** **       ** ***** ** ***** ** ***

TaGT2L1A         GTCAGTGCCGCCCCCCCGCCGCAGATGCACGCCTTCTCCGCGCCGCAGCCGATGAGCGCA
TaGT2L1B         GTCAGTGCAGCCCCCCCGCCGCAGATGCACGCCTTCTCGGCGCCCCAGCCGATGAGCGCA
TaGT2L1D         GTCAGTGCCGCCCCCCCGCCGCAGATGCACGCCTTCTCCGCGCCGCAGCCGATGAGCGCA
                 ******** ***************************** ***** ***************

TaGT2L1A         ATGCCGCCGCCGCCGGGGCCGATGCAGCCGGCCCCTATATCTTCGGCGGCGCCTGCGCCG
TaGT2L1B         ATGCCGCCGCCGCCGGGGCCGATGCAGCCGGCCCCTATATCTTCGGCGGCCCCTGCGCCG
TaGT2L1D         ATGCCGCCGCCGCCGGGGCCGATGCAGCCGGCCCCTATATCTTCGGCCGCCCCTGCGCCG
                 *********************************************** ** *********

TaGT2L1A         GCCGTGGAGCCTCCCCAGCCGCCGCCTGTCAGCCTGCAAGGTCTGAGCTTCCCGTGCATG
TaGT2L1B         GTCGTGGAGCCTCCCCATCCGCTGCCTGTCAGCCTGCAAGGCCTCAGCTTCCCGTCCATG
TaGT2L1D         GCCGTGGAGCCTCCCCAGCCGCCGCCTGTCAGCCTGCAAGGCTTGAGCTTCCCGTCCATG
                 * *************** **** ******************  * ********** ****

TaGT2L1A         TCCGACTCCGAGTCGGATGACGACGACGACTCCGAGGACGACGACATGACGGCCGAGACC
TaGT2L1B         TCCGACTCTGAGTCGGACGACGACGACGACTCCGAGGATGACGACATGACGGCCGAGACA
TaGT2L1D         TCCGACTCTGAGTCGGACGACGACGACGACTCCGAGGACGACGACATGACGGCCGAGACC
                 ******** ******** ******************** ******************** 

TaGT2L1A         GGCGGCAGCCCGGACGGCCTCGGCAAGCGAAAGCGTGGCGGCGGGGGTAGCAAAAAGATG
TaGT2L1B         GGCGGCAGCCCGGATGGCCTCGGCAAGCGCAAGCGCGGCGGCGGCGGCAGCAAGAAGATG
TaGT2L1D         GGCGGCAGCCCGGATGGCCTCGGCAAGCGCAAGCGCGGCGGCGGCGGCAGCAAGAAGATG
                 ************** ************** ***** ******** ** ***** ******

TaGT2L1A         ATGGCTTTCTTCGAGGGCCTGATGAAGCAGGTCGTACAGAGGCAGGAGGAGATGCAGCAG
TaGT2L1B         ATGGCTTTCTTCGAGGGCCTGATGAAGCAGGTCGTACAGAGGCAGGAGGAGATGCAGCAG
TaGT2L1D         ATGGCTTTCTTCGAGGGCCTGATGAAGCAGGTCGTACAGAGGCAGGAGGAGATGCAGCAG
                 ************************************************************

TaGT2L1A         CGGTTCCTGGAGACCATGGAGAAGAGGGAGGCCGAGCGCACGGCGCGGGAGGAGGCCTGG
TaGT2L1B         CGGTTCCTGGAGACCATGGAGAAGAGGGAGGCCGAGCGCACGGAGCGGGAGGAGGCCTGG
TaGT2L1D         CGGTTCCTGGAGACCATGGAGAAGAGGGAGGCCGAGCGCACGGCGCGGGAGGAGGCCTGG
                 ******************************************* ****************

TaGT2L1A         CGCCGGCAGGAGGTGGCCCGGCTTAACCGTGAGCAGGAGCAGCTCGCCCAAGAGCGCGCC
TaGT2L1B         CGCCGGCAGGAGGTGGCCCGCCTTAACCGCGAGCAGGAGCAGCTCGCGCAGGAGCGCGCC
```

```
TaGT2L1D        CGCCGGCAGGAGGTGGCCCGGCTTAACCGCGAGCAGGAGCAGCTCGCCCAAGAGCGCGCA
                ******************** ******** ***************** ** ********

TaGT2L1A        GCGGCAGCTTCCCGCGATGCCTCCATCATCGCCTTCCTCCAGCGCATCGGTGGGCAGGCC
TaGT2L1B        GCGGCAGCTTCCCGCGATGCCTCCATCATCGCCTTCCTCCAGCGCATCGGTGGTCAGACC
TaGT2L1D        GCGGCGGCCTCCCGCGACGCCTCCATCATCGCCTTCCTCCAGCGCATCGGTGGGCAGACC
                ***** ** ******** *********************************** *** **

TaGT2L1A        GTGCATGTCCCACCCGTCGTCATCCCCATGCCAACGCCGATGCAGGTCCAGACCTCGCCG
TaGT2L1B        GTGCATGTCCCACCCGTCGTCATCCCCATGCCAACGCCGATGCAGGTCCAGACCCCGCCA
TaGT2L1D        GTGCATGTCCCACCCGTCGTCATGCCCATGCCAACGCCGATGCAGGTCCAGACCCCGCCG
                *********************** ****************************** ****

TaGT2L1A        CCGCCAAAGAAACCTCGACAGCACCTGCCGCCACCGCCACCGTCACAGGCCACACAGCAG
TaGT2L1B        CCGCCAAAGAAACCTCGGCAGCACCTGCCGCCACCGCCGCCGTCACAGGCCACACAGCAG
TaGT2L1D        CCGCCAAAGAAACCTCGACAGCACCTCCCACCCCCGCCGCCGTCACAGGCTACACAGCAG
                ***************** ******** ** ** ***** *********** *********

TaGT2L1A        CCACAGCCGCAGCCGCAGCCTCAGCCTCAGCCCATCCCAGCTGCGCCCCTCCAACAACAA
TaGT2L1B        CCGCAGCCGCAGCCGCAGCCG------CAACCCATCCCAGCTGCGCCGCTCCAACAGCAA
TaGT2L1D        CCGCAGC------CGCAGCCT------CAGCCCATCCCAGCTGCGCCCCTCCAACAGCAA
                ** ****      *******       ** ***************** ******** ***

TaGT2L1A        CCGCC---GTCGCAACCACCACACAAGGAAACGACGCGCGAAGAGGCCGGGACGCCCCGC
TaGT2L1B        CCGCCGCCGCAACCACCGCAGCACAAGGAAACGACGCGCGAAGAGGCCGGAACGCCCCGC
TaGT2L1D        CCGCCGCCGCAACCACCGCAGCACAAGGAAACGACGCGCGAAGAGGTCGGGACGCCCCGC
                *****   *   * *** *  ************************* *** *********

TaGT2L1A        AGCGCACCGGCCCCGACTTCTGCCGGCTTATCACTCGCGCTGGTTCCCGTCGCCACGGAG
TaGT2L1B        AGCGCACCGGCCCCGACTTCTGCCGGCTTATCACTCGCGCTGGTTCCCGTCGCCACGGAG
TaGT2L1D        AGCGCACCGGCCCCGACTTCTGCCGGCTTATCACTCGCGCTGGTTCCCGTCGCCACGGAG
                ************************************************************

TaGT2L1A        CAGCACGTCGTCGAGGCCGCTGGTCTGGGAGGAGGGGAGAGCGTAGGGGGGCCGTCGTCG
TaGT2L1B        CAGCACGTCGTCGAGGCCGCTGGTCTGGGAGGAGGGGAGAGCGGAGGGGGGCCGTCGTCG
TaGT2L1D        CAGCACGTCGTCGAGGCCGCTGGTCTGGGAGGAGGGGAGAGCGGAGGGGGGCCGTCGTCG
                ******************************************* ****************

TaGT2L1A        TCACGGTGGCCCAAGACGGAGGTGCACGCGCTTATCCAGCTGCGCATGGACATGGACAAT
TaGT2L1B        TCACGGTGGCCCAAGACGGAGGTGCACGCGCTTATCCAGCTCCGCATGGACATGGACAAT
TaGT2L1D        TCACGGTGGCCCAAGACGGAGGTGCACGCGCTTATCCAGCTCCGCATGGACATGGACAAT
                ***************************************** ******************

TaGT2L1A        CGCTACCAGGAGAACGGACCAAAGGGCCCGCTCTGGGAGGAGATCTCCGCCGGGATGCGG
```

```
TaGT2L1B        CGCTACCAGGAGAACGGACCAAAGGGCCCGCTGTGGGAGGAGATCTCCGCCGGGATGCGG
TaGT2L1D        CGCTACCAGGAGAACGGACCAAAGGGCCCGCTGTGGGAGGAGATCTCCGCCGGGATGCGG
                ******************************** ***************************

TaGT2L1A        CGGCTGGGTTACAGCCGCAACTCCAAACGGTGCAAGGAGAAGTGGGAGAACATCAACAAG
TaGT2L1B        CGGCTGGGTTACAGCCGCAACTCCAAACGGTGCAAGGAGAAGTGGGAGAACATCAACAAG
TaGT2L1D        CGGCTGGGTTACAGCCGCAACTCCAAACGGTGCAAGGAGAAGTGGGAGAACATCAACAAG
                ************************************************************

TaGT2L1A        TACTTCAAGAAGGTGAAGGAGAGCAACAAGAGGCGGCCGGAGGACTCCAAGACATGCCCG
TaGT2L1B        TACTTCAAGAAGGTGAAGGAGAGCAACAAGAGGCGGCCGGAGGACTCCAAGACATGCCCG
TaGT2L1D        TACTTCAAGAAGGTGAAGGAGAGCAACAAGAGGCGGCCGGAGGACTCCAAGACATGCCCG
                ************************************************************

TaGT2L1A        TACTTCCACCAGCTCGAGGCCATCTACCGCAAGAAGCACAACGGCGGCGGCAGCAGCGGC
TaGT2L1B        TACTTCCACCAGCTCGAGGCCATCTACCGCAAGAAGCACAACGGCAGCGGCAGCAGCGGC
TaGT2L1D        TACTTCCACCAGCTCGAGGCCATCTACCGCAAGAAGCACAACGGCGGCGGCAGCAGCGGC
                ********************************************* **************

TaGT2L1A        GCCGCGGCCAACAACGCCGTCGTGCCTGTCCCTGCCGTCGCGGAGCATCAGAACCTGAAC
TaGT2L1B        GCCGCGGCCAACAACGCCGTCGTGTCTGTCCCTGCCGTCGCGGAGCATCAGAACCTGAAC
TaGT2L1D        GCCGCGGCCAACAACGCCGTCGTGTCTGTCCCTGCCGTCGCGGAGCATCAGAACCTGAAC
                ************************ ***********************************

TaGT2L1A        CGGCACGAGATCGAGATCGAGGGGAAGAAGATCAACGACACCGACAAGAGGAACAACGGA
TaGT2L1B        CGGCACGAGATCGAGATCGAGGGGAAGAAGATCAACGACACCGACAAGAGGAACAACGGA
TaGT2L1D        CGGCACGAGATCGAGATCGAGGGGAAGAAGATCAACGACACCGACAAGAGGAACAACGGA
                ************************************************************

TaGT2L1A        GGAGTCGGAGCCGCGCAGGTACCGACAAGCAACGGGCAGACAACGCCGCCGACGGCCACG
TaGT2L1B        GGAGTCGGAGCCGCGCAGGTGCCGACAAGCAACGGGCAGACAACGCCGACGACGGCCACG
TaGT2L1D        GGAGTCGGAGCCGCGCAGGTGCCGACAAGCAACGGGCAGACAACGCCGACGATGGCCACG
                ******************** *************************** *** *******

TaGT2L1A        TTCGACCTGGGTGTAAAAAAGCCAGAAGACGCTGTGAGGGAGCTGAACGAGCAGCCGCAC
TaGT2L1B        TTCGACCTGGGCGTAAAAAAGCCAGAAGACGCTGTGAGGGAGCTGAACGAGCAGCCGCAC
TaGT2L1D        TTCGACCTGGGCGTAAAAAAGCCAGAAGACGCTGTGAGGGAGCTGAACGAGCAGCCGCAC
                *********** ************************************************

TaGT2L1A        CGGGAGTTCACGACGGACGAGACCGACAGCGACGACATGGGCGATGACTACACTGACGAC
TaGT2L1B        CGGGAGTTCACGACGGACGAGACCGACAGCGACGACATGGGCGATGACTACACTGACGAC
TaGT2L1D        CGGGAGTTCACGACGGACGAGACCGACAGCGACGACATGGGCGATGACTACACTGACGAC
                ************************************************************
```

```
TaGT2L1A        GGCGAGGACGGCGAGGACGACGGCAAAATGCAGTACAGGATACAGTTCCAGAGGCCAAAC
TaGT2L1B        GGCGAGGACGGCGAGGACGACGGCAAAATGCAGTACAGGATACAGTTCCAGAGGCCAAAC
TaGT2L1D        GGCGAGGACGGCGAGGACGACGGCAAAATGCAGTACAGGATACAGTTCCAGAGGCCAAAC
                ************************************************************

TaGT2L1A        CCCGTCGGCACCAACAATGCGCCTCCATCACCAACCACCGCGGCGACAGCGGCGCCGACA
TaGT2L1B        CCCGTCGGCACCAACAATGCGCCTCCACCGCCAACCACCGCGGCGACAGCAGCGCCGACA
TaGT2L1D        CCGGTCGGCACCAACAATGCGCCTCCACCGCCAACCACCGCGGCGACAGCAGCGCCGACA
                ** ************************ * ******************** *********

TaGT2L1A        TCGACCCCCGCGAGCTCCTTTCTCGCCATGGTTCAG
TaGT2L1B        TCGACCCCCGCGAGCTCCTTCCTCGCCATGGTTCAA
TaGT2L1D        TCGACCCCCGCGAGCTCCTTCCTCGCCATGGTTCAG
                ******************** ************** 
```

Supplementary Fig. S1 The nucleotide sequences of *TaGT2L1A*, *TaGT2L1B*, and *TaGT2L1D*. Sequences alignments were performed using Clustal Omega 1.2.1. The asterisks indicate the consensus sequences.

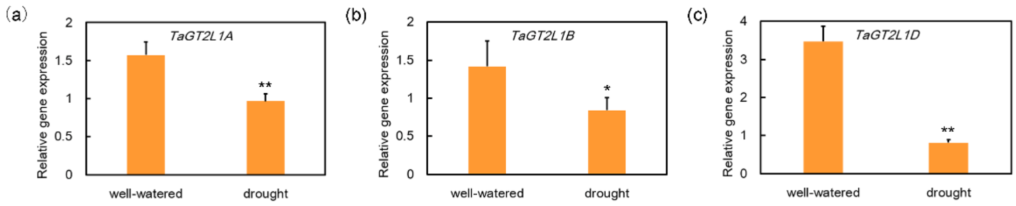

Supplementary Fig. S2 Drought treatment suppressed the expression of *TaGT2L1A*, *TaGT2L1B*, and *TaGT2L1D*. One-week-old wheat seedlings were cultured with (well-watered) or without (drought) watering for three weeks, and then total RNA was isolated from leaf samples and reverse-transcribed (n = 6). The qPCR was performed to determine the expression of *TaGT2L1A* (**a**), *TaGT2L1B* (**b**), and *TaGT2L1D* (**c**) using *TaACTIN* gene as an internal reference. All values are the means ± standard deviations of three independent experiments. Asterisks represent statistically significant differences compared with well-watered group (Student's *t*-test, *P < 0.05, **P < 0.01).

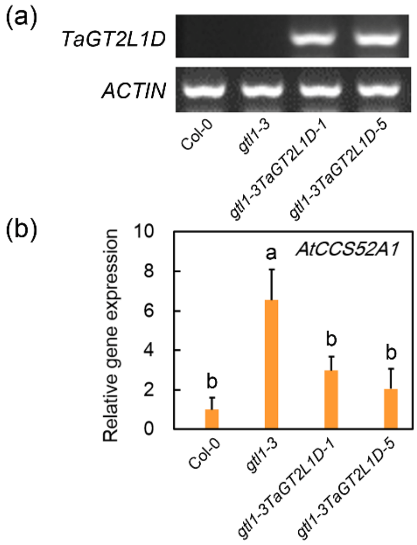

Supplementary Fig. S3 Expression levels of *TaGT2L1D* and *AtCCS52A1* in Col-0, *gtl1-3*, and gtl1-3 TaGT2L1D lines. Total RNA from six-week-old Col-0, *gtl1-3*, *gtl1-3TaGT2L1D-1*, and *gtl1-3TaGT2L1D-5* plants was isolated and reverse-transcribed (n = 6). Gene-specific primers were used to examine the expression of *TaGT2L1D* (**a**) and *AtCCS52A1* (**b**). RT-PCR and qPCR were performed using *AtACTIN* as an internal reference. All values are the means ± standard deviations of three independent experiments. Different letters indicate a significant differences (Student-Newman-Keuls test, $P < 0.05$).

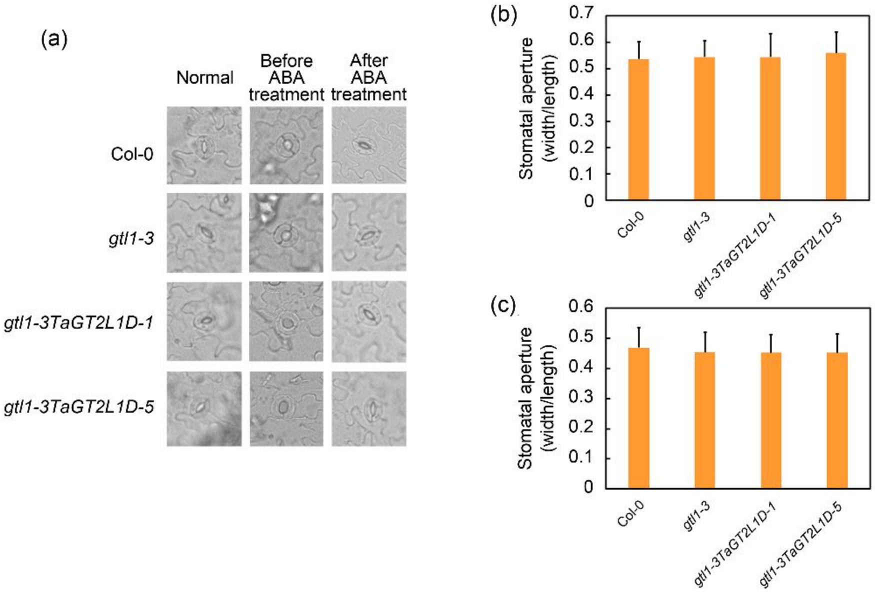

Supplementary Fig. S4 Stomatal aperture in wild-type Col-0, *gtl1-3*, and *gtl1-3TaGT2L1D* lines under normal condition or ABA treatment. (**a**) Stomatal apertures in Col-0, *gtl1-3*, *gtl1-3TaGT2L1D-1*, and *gtl1-3TaGT2L1D-5* plants under normal condition or after 20 μM ABA treatment for 1 h. "Normal" indicates stomatal apertures in normal condition; "Before ABA treatment" indicates stomatal apertures after 2 h incubation in a buffer solution (20 mM KCl, 5 mM MES-KOH, and 1 mM $CaCl_2$, pH 6.15); "After ABA treatment" indicates stomatal apertures after 1 h 20 μM ABA incubation. Representative micrographs are shown. (**b**) Stomatal apertures of Col-0, *gtl1-3*, *gtl1-3TaGT2L1D-1*, and *gtl1-3TaGT2L1D-5* plants corresponding to "Normal" group in (**a**). (**c**) Stomatal apertures of Col-0, *gtl1-3*, *gtl1-3TaGT2L1D-1*, and *gtl1-3TaGT2L1D-5* plants corresponding to "After ABA treatment" group in (**a**). All values are the mean ratios of width to length ± standard deviations of three independent experiments (n = 60).

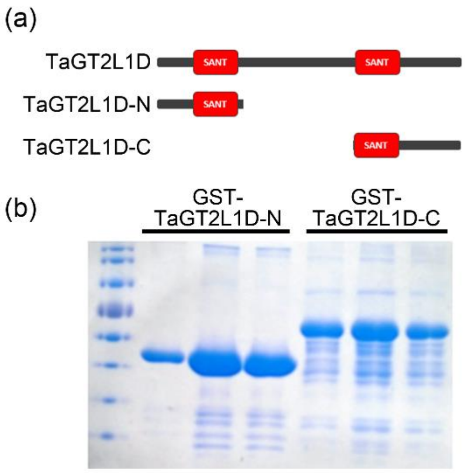

Supplementary Fig. S5 Expression and purification of GST-TaGT2L1D-N and GST-TaGT2L1D-C proteins. (**a**) Schematic diagrams of TaGT2L1D-N and TaGT2L1D-C proteins. Red boxes indicate SANT domains. (**b**) The purified proteins were separated on a 10% polyacrylamide gel, and Coomassie brilliant blue (CBB) staining was performed to show the purity of GST-TaGT2L1D-N and GST-TaGT2L1D-C proteins.

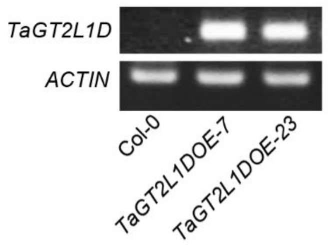

Supplementary Fig. S6 Expression level of *TaGT2L1D* in the *TaGT2L1DOE* lines. Total RNA from four-week-old Col-0, *TaGT2L1DOE-7*, and *TaGT2L1DOE-23* plants was isolated and reverse-transcribed (n = 6). RT-PCR was performed using *AtACTIN* as an internal reference.
